# Probiotic-loaded seed mucilage-based edible coatings for fresh pistachio fruit preservation: an experimental and modeling study

**DOI:** 10.1038/s41598-023-51129-6

**Published:** 2024-01-04

**Authors:** Aref Zibaei-Rad, Mostafa Rahmati-Joneidabad, Behrooz Alizadeh Behbahani, Morteza Taki

**Affiliations:** 1 Department of Horticultural Science, Faculty of Agriculture, Agricultural Sciences and Natural Resources University of Khuzestan, P.O. Box: 6341773637, Mollasani, Iran; 2 Department of Food Science and Technology, Faculty of Animal Science and Food Technology, Agricultural Sciences and Natural Resources University of Khuzestan, P.O. Box: 6341773637, Mollasani, Iran; 3 Department of Agricultural Machinery and Mechanization Engineering, Faculty of Agricultural Engineering and Rural Development, Agricultural Sciences and Natural Resources University of Khuzestan, P.O. Box: 6341773637, Mollasani, Iran

**Keywords:** Antimicrobials, Pathogens

## Abstract

In this study, *Lallemantia royleana* mucilage (LRM) based edible coating containing 1.5 × 10^8^ and 3 × 10^9^ CFU/mL *Lacticaseibacillus casei* XN18 (Lbc1.5 and Lbc3) was designed to improve the quality and shelf-life of fresh pistachio. The fresh pistachios were coated with LRM + Lbc and their physicochemical, microbial, and sensory properties were evaluated after 1-, 5-, 15-, 25-, and 35-day storage at 4 °C. By the end of storage day, in comparison to control, the presence of probiotic isolate in the edible coating (particularly LRM + Lbc3) led to a marked decrease in fungal growth (3.1 vs. 5.8 Log CFU/g), weight loss (6.7 vs. 8.1%), and fat oxidation (0.19 vs. 0.98 meq O_2_/kg), and preserved total chlorophylls (8.1 vs. 5.85 mg/kg) and phenols (31.5 vs. 20.32 mg GAE/100 g), and antioxidant activity (38.95 vs. 15.18%) of samples during storage period. Furthermore, LRM + Lbc3-coated samples had a probiotic number above the recommended level (6.85–9.29 log CFU/g) throughout storage. The pistachios coated with probiotic-enriched edible coatings were greatly accepted by panelists. In the next section, Gaussian Process Regression (GPR) was used for predicting some parameters including: weight loss, TSS, Fat content, PV, Soluble carbohydrate content, Viability, Total phenolic compounds, Antioxidant activity, Mold and yeast, Total chlorophylls, Total carotenoids, Color, Odor and Overall acceptance. The results indicated that, there is a good agreement between the actual and predicted data by GPR model and it can be used for similar situation to decrease the cost of laboratory tests and increase the respond of analysis.

## Introduction

As a healthy snack, pistachio (*Pistacia vera* L.) nuts are high in phenolics, essential fatty acids and minerals, vitamin A, and protein. Its dried shelled edible part, the kernel/nut, is routinely marketed around the world; however, fresh pistachios are also being consumed due to their high nutritional value, antioxidant phytochemicals, and unique taste^1^. Nonetheless, physiological and biochemical changes in fresh pistachios, as well as the growth of microorganisms, can reduce their shelf life. Moreover, fresh pistachios can undergo microbial spoilage, weight loss, texture softening, and changes in the nutrients, surface color, and organoleptic properties during storage^2^. It has been found that edible coatings can solve this problem^1,3^.

The application of edible coatings is an effective postharvest strategy to minimize quality loss during storage, including browning^4–6^. As edible coatings create an air barrier between products and the atmosphere, they reduce water loss and gas exchange, which in turn reduces fruit respiration and moisture loss. In addition to maintaining product quality, edible coatings can also reduce microbial growth by creating a modified atmosphere around the product^7–9^. In this regard, the edible coatings used in Javanmard's study reduced oxidation and improved shelf life of pistachio kernels^10^.

Mucilage, a long-chain polysaccharide substance, is applied in the food industry as a food additive and gelling agent. The Shirazi balangu (*Lallemantia royleana*) belongs to the *Lamiaceae* family and is grown throughout Iran as a medicinal plant. Various therapeutic effects of *L. royleana* seeds have been reported, including treatment of inflammation, respiratory problems, and abscesses. Seeds readily swell in water and release a large amount of mucilage (soluble gum). *L. royleana* seed mucilage (LRM), a local Iranian hydrocolloid, is used in the production of edible coatings and formulations in the food industry^11,12^. Antimicrobial and antioxidant agents are often added to edible coatings to improve their effectiveness^7,9,12^.

The gut microbiota is known to play an important role in supporting health by enhancing nutrient absorption, host resistance against infection, and the immune system of the intestine^13^. Approximately 100 trillion microorganisms play a crucial role in maintaining gastrointestinal homeostasis within the human body, according to a recent study^14^. The presence of probiotic bacteria improves the microflora in the intestine, maintaining a balanced eubiosis. Probiotics such as *Lactobacillus* and *Bifidobacterium* are the most commonly used by humans^15^. Various beneficial properties of probiotics bacteria are triggered by their consumption, including masking the pathogens' binding sites and inhibiting their colonization, stimulating the immune system, lowering blood cholesterol, treating vaginal yeast infections, alleviating the symptoms of irritable bowel syndrome and colitis, reducing dental caries, preventing various cancers such as colorectal, and helping with weight loss^13,16^. The Gram-positive *Lacticaseibacillus casei* (former *Lactobacillus casei*) is a facultative heterofermentative lactobacillus found in fermented dairy, vegetable, and cereal products, as well as in the gastrointestinal tracts of humans and animals. It is extensively studied because of its industrial value and health-promoting potential, including the ability to suppress tumors, alleviate inflammation, and improve and regulate intestinal microbiota^17^. Several studies used probiotic microorganisms with antimicrobial and antioxidant activity as biocontrol agents^16,18,19^. Therefore, it is expected that the use of an edible coating based on mucilage and probiotics, in addition to preserving the physicochemical and sensory characteristics and reducing mold growth on the product surface, can significantly improve its health-promoting properties.

Simulation and mathematical modeling of qualitative properties of foods are one of the subjects that recently used by some researchers in food subjects^20^. The purpose of modeling is to choose the best method and create the most accurate output data. Sometimes, it is not possible to conduct laboratory tests and configure the system because of high cost. So, in the food industry, modeling application to predict the qualitative properties of foods can be very useful and is known as a valuable tool^21^. Mathematical and machine learning models and simulation techniques such as Artificial Intelligence (AI), fuzzy, Gaussian Process Regression etc., can be effectively used to predict and analyze the effect of changes in input variables and its effects on outputs. Some researchers applied intelligent models for qualitative properties of foods^22–24^.

Nevertheless, no research has been conducted using LRM edible coating combined with *Lb. casei* XN18 (Lbc XN18) on fresh pistachios. Due to the poor storage stability of fresh pistachios, this study investigated how probiotic coating affects quality attributes during cold storage.

## Materials and methods

### Materials

Fresh pistachio fruit cv. ‘Ahmad-Aghaei’ was manually harvested from healthy pistachio trees (Torbat-e-Jam, Khorasan Razavi, Iran). It is indeed important to mention that the collection of fresh pistachio fruit was done by the first author of the article (Aref Zibaei-Rad) from his personal garden (Aref Zibaei-Rad is the owner of the garden). This clarification accurately attributes the collection process to the first author and acknowledges their role in obtaining the samples. It is important to mention that the identification of the fresh pistachio fruit was carried out by Dr. Kazem Negaresh at the herbarium center of Agricultural Sciences and Natural Resources University of Khuzestan. Furthermore, it is reiterated that the experimental field study was conducted in accordance with applicable institutional guidelines and regulations, ensuring compliance with ethical standards. This statement underscores the commitment to ethical practices and adherence to the necessary protocols during the study. This study used fruits without cracks, with uniform sizes, colors, and shapes, immediately after harvesting. *L. royleana* seeds were purchased from a local market (Mashhad, Iran). Other chemicals were of analytical grade and purchased from Merck Co. (Germany) or Sigma-Aldrich Co. (USA).

### Edible coating preparation and pistachio coating

The LRM plant was obtained from its natural habitat in Mashhad, Razavi Khorasan province, Iran. The identification of the plant was carried out by Dr. Kazem Negaresh at the herbarium center of Agricultural Sciences and Natural Resources University of Khuzestan, with the herbarium code KHAU. The experimental field study was conducted in accordance with applicable institutional guidelines and regulations, ensuring compliance with ethical standards. LRM was extracted according to Alizadeh Behbahani and Imani Fooladi method^11^. It was blended with Tween 80 (35% of the LRSM dry weight), dissolved in distilled water (100 mL), heated, and then cooled down to room temperature. The probiotic strain (*Lb. casei* XN18) was then added to the polymeric solution at 1.5 × 10^8^ CFU/mL (Lbc1.5) and 3 × 10^9^ CFU/mL (Lbc3) concentrations to prepare probiotic edible coatings.

The fresh pistachio samples were coated with LRM, LRM + Lbc1.5, and LRM + Lbc3, and air-dried for 60 min at room temperature. As a final step, the samples were packaged in polyethylene plastic bags (150 g each) in three replications, and stored at 85% relative humidity and 4 °C for up to 35 days. The non-coated pistachio sample was used as control^1,25^.

### Fungi count

The count of mold and yeast was determined by pouring diluted sample (0.1 mL; 10^–2^ and 10^–3^) onto potato dextrose agar plates containing 0.1 g/L chloramphenicol. Incubation at 25 °C for 5 days was followed by colony counting^26^.

### Weight loss

The method of Tano et al. was applied to measure the weight loss (%) of samples during storage. It was determined by monitoring the weight of the contents of the package before and after storage^27^.

### Total soluble solid (TSS) and soluble carbohydrate content (SCC)

A digital refractometer was used to assess the TSS value fruit extracts. The amount of soluble carbohydrates was measured by sulfuric acid-phenol method. Briefly, the absorbance of sample extracts was read at 485 nm, and the content of soluble carbohydrate contents (%) was determined using a standard curve based on glucose^28^.

### Fat content and peroxide value (PV)

The fat content of the pistachio was determined according to the method of Khajeh-Ali et al.^25^. The PV of the extracted oil was determined using titration method as described by Hashemi et al.^1^, and expressed as meq O_2_/kg oil.

### Total chlorophylls and carotenoids content

Nazoori et al.^29^ method was used to measure the total chlorophylls and carotenoids content. Briefly, the sample was ground in 80% acetone (in water) with a mortar and pestle and the obtained mixture was centrifuged (5000 rpm, 20 min). Then, 80% acetone was added to the supernatant, and its absorbance was recorded at 470, 646.8 and 663.2 nm. The concentrations of total chlorophylls and carotenoids were expressed as mg/kg.

### Total phenol contents (TPC)

Pistachio hulls (500 mg) were crushed and mixed with 3 mL of 85% methanol (3 ml, 85%), and then centrifuged (12,000×*g,* 20 min). The obtained supernatant (300 μL) was mixed with sodium carbonate (7%; 1200 μL) and Folin-Ciocalteu reagent (10%; 1500 μL), and shaken for 90 min at room temperature in a dark place. The absorbance of the solution was measured at 760 nm, and TPC of the samples was calculated using gallic acid as external standard^1^.

### Antioxidant activity

The DPPH radical scavenging activity of the samples was evaluated using Ehteshami et al.^30^ with some modification. To do this, 0.1 mL of the hull extract was mixed with 1 mL of Tris–HCl buffer (pH 7.5), and 1 mL of 0.1 mM DPPH. The mixture was stored in a dark place for 30 min, and its absorbance was read at 517 nm:

$$\text{Antioxidant activity }\left(\text{\%}\right) = \left(1-\frac{\text{ Abs sample}}{\text{Abs control}}\right) \times {100}$$

### Probiotic viability

Pistachio samples were tested for survivability of the strain during storage period. Randomly selected fruits (25 g) were blended with saline solution (225 mL) in stomacher bags. For strain enumeration, tenfold serial dilutions were plated onto CaCO_3_-loaded MRS agar and incubated at 37 °C for 48 h, and results were expressed as log CFU/g^18^.

### Sensory evaluation

The evaluation of the acceptance of sensory attributes (color, aroma, and overall acceptance) was conducted using a nine-point hedonic scale (1 = disliked extremely and = liked extremely). To code the formulations, three-digit numbers were used, and 25 judges participated in the sensory panel^16^.

### Statistical analysis

Experiments were repeated three times. Analyses were conducted with Minitab software (version 19). The Tukey test (*p* < 0.05) was used to determine whether there were significant differences between the means.

### Gaussian process regression (GPR) model

**Figure 1:**
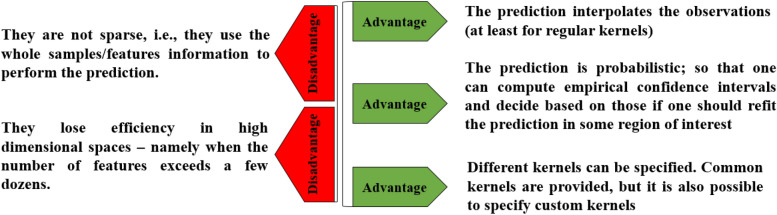
Advantages and disadvantages of GPR model.

Gaussian Process Regression (GPR) is a powerful class of machine learning algorithms that rely on a few parameters to make predictions, unlike many of machine learning models. Because GPR is (nearly) nonparametric, it can effectively solve a variety of supervised learning problems even with little data^31^. Figure 1 shows the all advantages and disadvantages of GPR model.

GPR model takes the input as training data to create a model that popularizes well to the distribution of the output at unseen input locations^21^. GPR works under the probabilistic regression framework, which takes as input a training data set ${\text{D = }}\left\{ {{\text{(y}}_{{\text{n}}} {\text{,x}}_{{\text{n}}} {\text{), n = 1,2,3,}}...{\text{,N}}} \right\}$ of N pairs of vector input $x_{n} \in R^{L}$ and noisy scalar output yn, and constructs a model that generalizes well to the distribution of the output at unseen input locations. The noise in the output models uncertainty due to factors external to x, such as truncation or observation errors. Here we assume that noise is additive, zero-mean, stationary and normally distributed, such that:

1 $$ y = f(x) + \varepsilon , \, \varepsilon \approx N(0,{\text{s}}_{noise}^{2} ) $$

where $s_{noise}^{2}$ is the variance of the noise^31^. The noise in the output model is the ambiguity due to element other than x, such as observation error. The consistency requirement allows us to use a set of training data to derive function values^21^. The function can be readable by the mean *m(x)* and covariance ${\text{k}} (x,x^{\prime})$ functions using the Gaussian prior assumption. The shape of the mean function is only matter in the unobserved regions and is usually set to zero^21^.

## Results and discussion

### Fungi count

**Figure 2:**
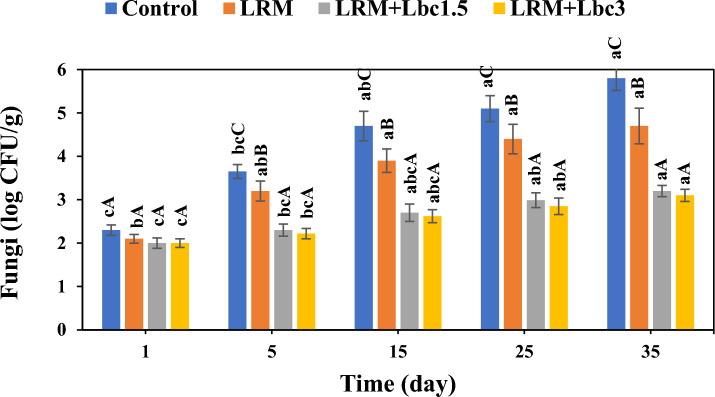
Changes in total fungi count of fresh in-hull pistachio samples coated with *Lallemantia royleana* mucilage (LRM) containing 1.5 × 10^8^ and 3 × 10^9^ CFU/ml *Lb. casei* XN18 (Lbc1.5 and Lbc3) during storage period. Significant small and capital letters indicate the effect of storage time and coating type on the response, respectively (p < 0.05).

The fungi count of pistachio samples during storage period is illustrated in Fig. 2. All samples showed a significant increase in fungi count as the storage time increased (p < 0.05). Nonetheless, the use of edible coating retarded the rise in microbial count markedly, and this effect was more pronounced when the probiotic cells were incorporated in the coating. At the end of storage time, the fungi count in the control, LRM, LRM + Lbc1.5, and LRM + Lbc3 coated pistachios were observed to be 5.8, 4.7, 3.2, and 3.1 log CFU/g, respectively. This means that the presence of the probiotic isolate in the edible coating resulted in a marked inhibition effect on the growth of fungal species on pistachio’s surfaces, mainly due to its antimicrobial effect^32^. Lactic acid bacteria, through their production of substances such as bacteriocins, organic acids, hydrogen peroxide, and diacetyl, have the ability to diminish the presence of pathogenic and spoilage-causing microorganisms in fresh fruit^19^. This would delay the fruit deterioration remarkably. Similar results have been reported by Temiz and Özdemir^19^, who worked on the effect of edible coating containing probiotic cells on the shelf-life of strawberries.

### Weight loss

**Figure 3:**
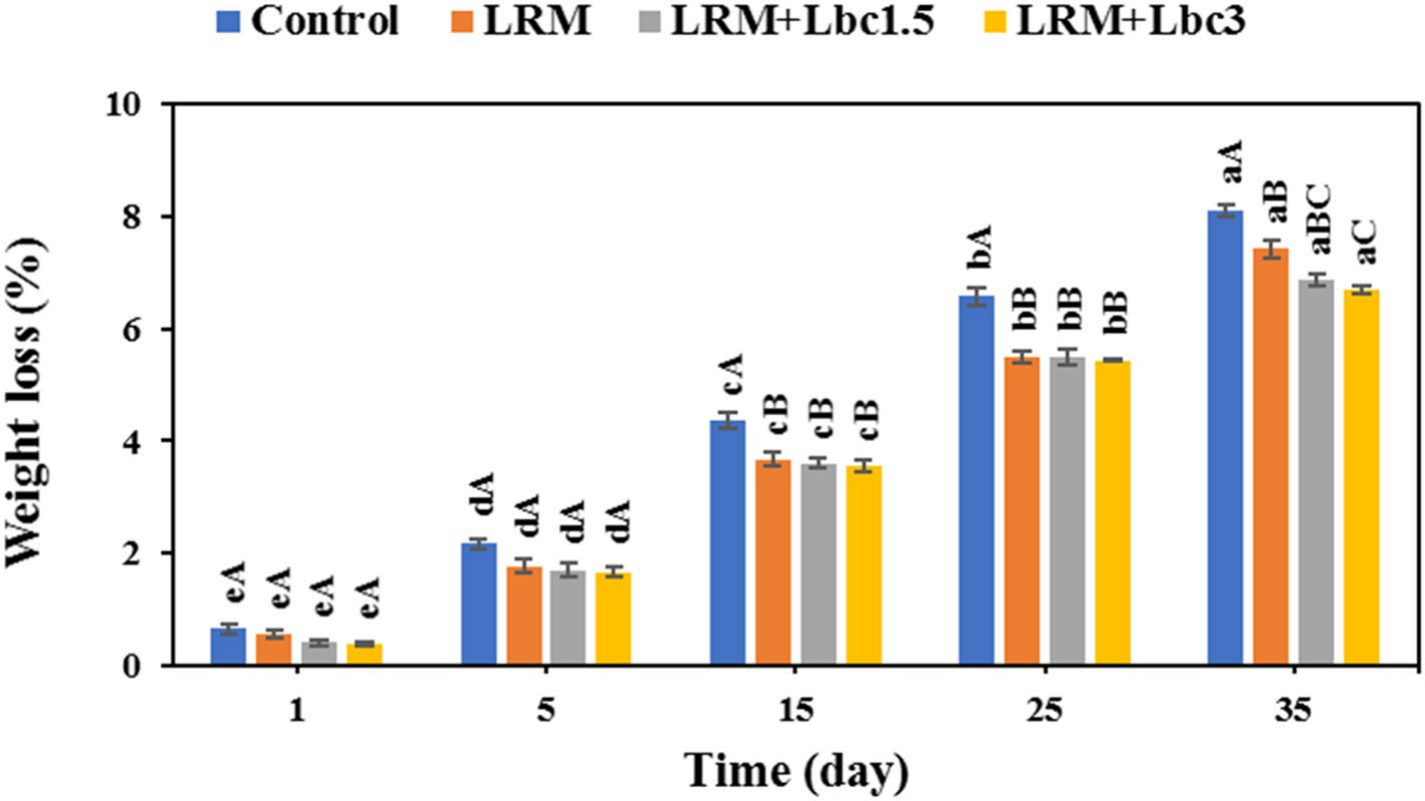
Changes in weight loss of fresh in-hull pistachio samples coated with *Lallemantia royleana* mucilage (LRM) containing 1.5 × 10^8^ and 3 × 10^9^ CFU/ml *Lb. casei* XN18 (Lbc1.5 and Lbc3) during storage period. Significant small and capital letters indicate the effect of storage time and coating type on the response, respectively (p < 0.05).

The changes in weight loss of samples are indicated in Fig. 3. As it can be observed, all samples experienced a significant increase in weight loss as a function of storage time (p < 0.05). The effect of sample type on the weight loss was also significant, the control sample had the highest weight loss changes (p < 0.05). The weight loss in control, LRM, LRM + Lbc1.5, and LRM + Lbc3 samples by the end of storage period was found to be 8.10, 7.42, 6.87, and 6.70%, respectively. The higher the probiotic cell in the coating, the lower was the pistachio’s weight loss. In line with our results, it has been reported that the dehydration and fruit shriveling can be retarded by coatings, which act as physical and moisture barriers^18^. Shayanfar et al.^26^ reported that the use of high barrier polyethylene bags provided a remarkably lower weight loss (< 0.5%) in fresh in-hull pistachios compared to the non-coated samples after 42 days of storage.

### TSS and SCC

**Figure 4:**
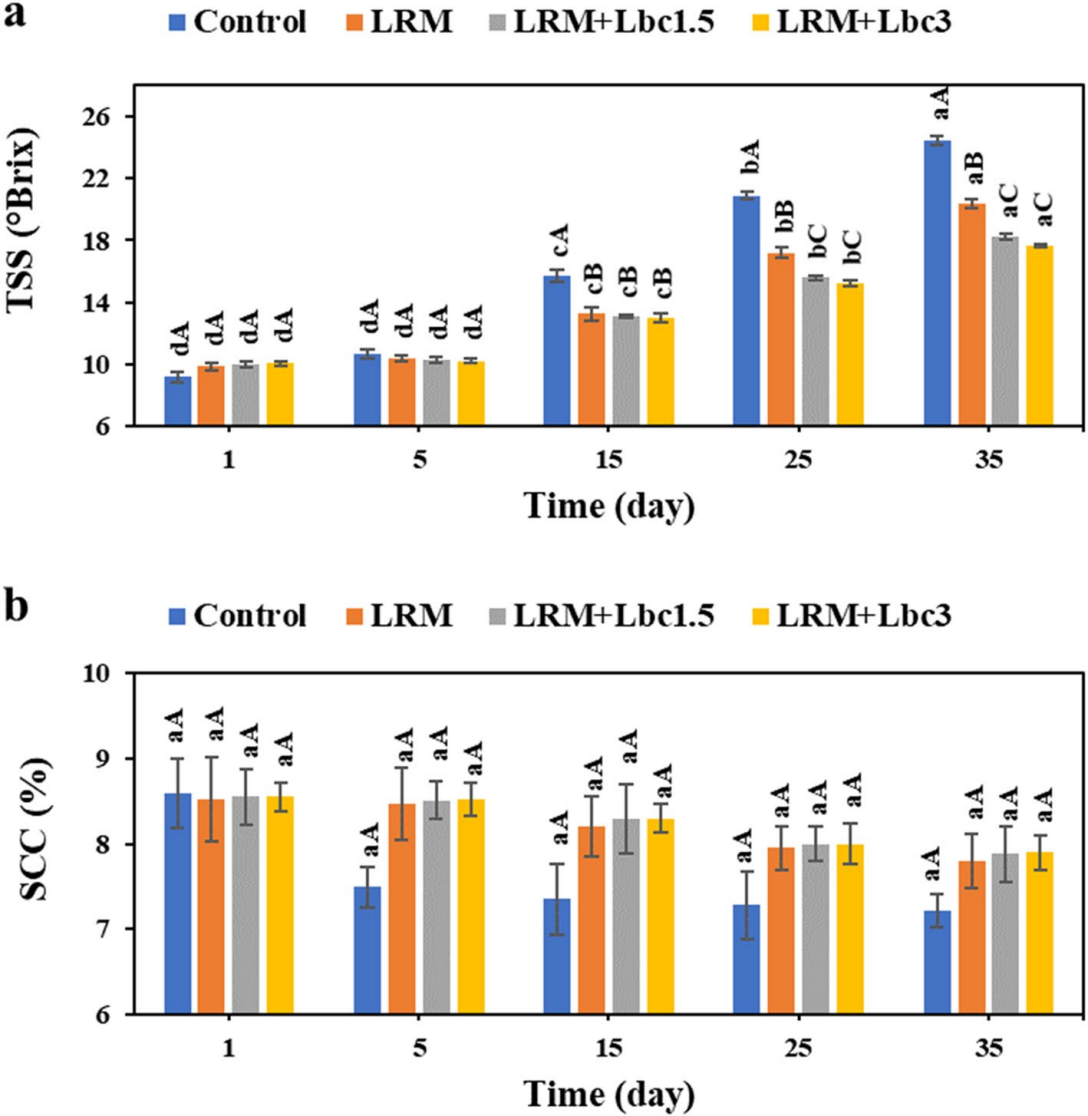
Changes in total soluble solids-TSS (**a**), and soluble carbohydrate content-SCC (**b**) of fresh in-hull pistachio samples coated with *Lallemantia royleana* mucilage (LRM) containing 1.5 × 10^8^ and 3 × 10^9^ CFU/ml *Lb. casei* XN18 (Lbc1.5 and Lbc3) during storage period. Significant small and capital letters indicate the effect of storage time and coating type on the response, respectively (p < 0.05).

The effect of edible coating on TSS and SCC of pistachio samples during storage is provided in Fig. 4. The TSS of samples increased significantly as the storage time increased, and the control sample had the highest TSS increase. The edible coatings containing probiotic cells were, however, able to inhibit the large changes in TSS. This might be ascribed to the lower weight loss and sugar consumption by fungal species (i.e., anti-fungal activity of the strain)^33^. The SCC of samples decreased non-significantly as a function of storage time (p > 0.05), and the highest and lowest SCC were accounted for control and LRM + Lbc3 during storage period. This could be due to the ability of edible coating in providing a controlled atmosphere around the sample and, in turn, lowering respiration rate and transpiration loss^34^. Similarly, Shakerardekani et al.^35^ reported that SCC of fresh pistachios decreased as a function of storage time; however, samples coated with sodium alginate enriched with thyme essential oil had remarkably higher SCC than the control pair.

### Fat content and peroxide value

**Figure 5:**
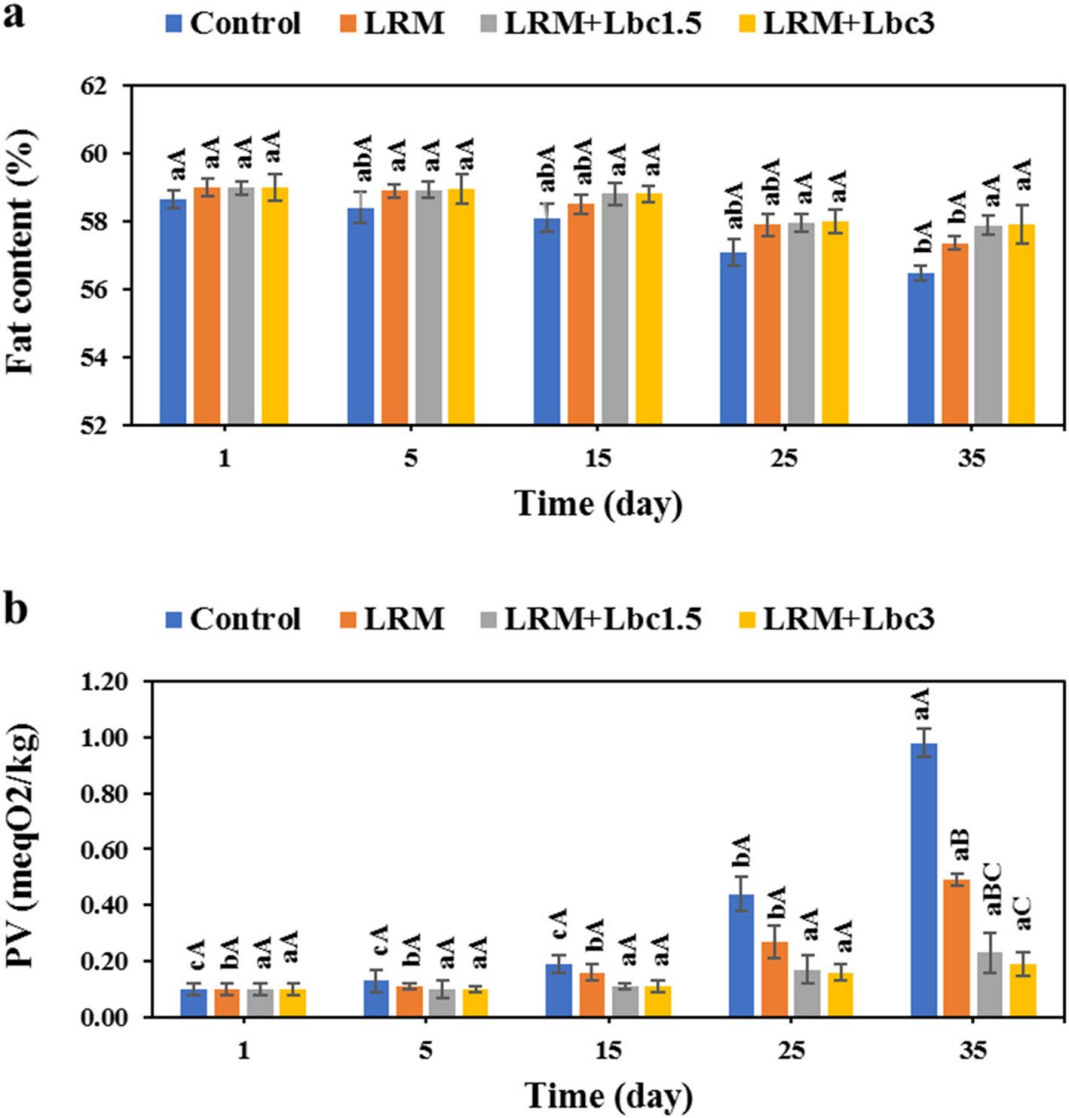
Changes in fat content (**a**) and peroxide value-PV (**b**) of fresh in-hull pistachio samples coated with *Lallemantia royleana* mucilage (LRM) containing 1.5 × 10^8^ and 3 × 10^9^ CFU/ml *Lb. casei* XN18 (Lbc1.5 and Lbc3) during storage period. Significant small and capital letters indicate the effect of storage time and coating type on the response, respectively (p < 0.05).

Pistachio oil (40–63%) is rich in polyunsaturated fatty acids such as linoleic and linolenic acids^36^. There was a significant decrease in fat content of control (58.66 to 56.49%) and LRM (58.98 to 57.36%) pistachios as the storage duration increased (Fig. 5a). The use of probiotic cells in LRM coating, however, inhibited the marked decrease in fat content of pistachio samples. After 35 days of storage, the fat content of LRM + Lbc1.5 and LRM + Lbc3 samples was significantly higher compared to the control and LRM counterparts. The changes in PV of non-coated and coated pistachios are indicated in Fig. 5b. As it can be seen, the PV of the samples increased over time, and the changes in PV of the control and LRM samples during storage period were significant (p < 0.05). And the lowest PV alterations were found in pistachios coated with LRM enriched with probiotic cells (p > 0.05). Generally, the PV of the samples at the end of storage time followed the order: control (0.98 meq O_2_/kg) > LRM (0.49 meq O_2_/kg) > LRM + Lbc1.5 (0.23 meq O_2_/kg) > LRM + Lbc3 (0.19 meq O_2_/kg). The higher fat content and lower PV in probiotic cell-loaded LRM coated pistachios could be due to the antioxidant activity of the probiotic strain and lower oxygen permeability of the edible coating^3^. The oxygen penetration into samples could be thus decreased, leading to a lower respiration rate, and fat decomposition and oxidation^2^. Similar findings were reported by Khoshnoudi-Nia and Sedaghat^3^ and Ahmadi et al.^28^, who demonstrated that coating pistachio samples with bioactive edible coating inhibited fat loss and oxidation of samples during storage.

### Total chlorophylls and carotenoids content

**Figure 6:**
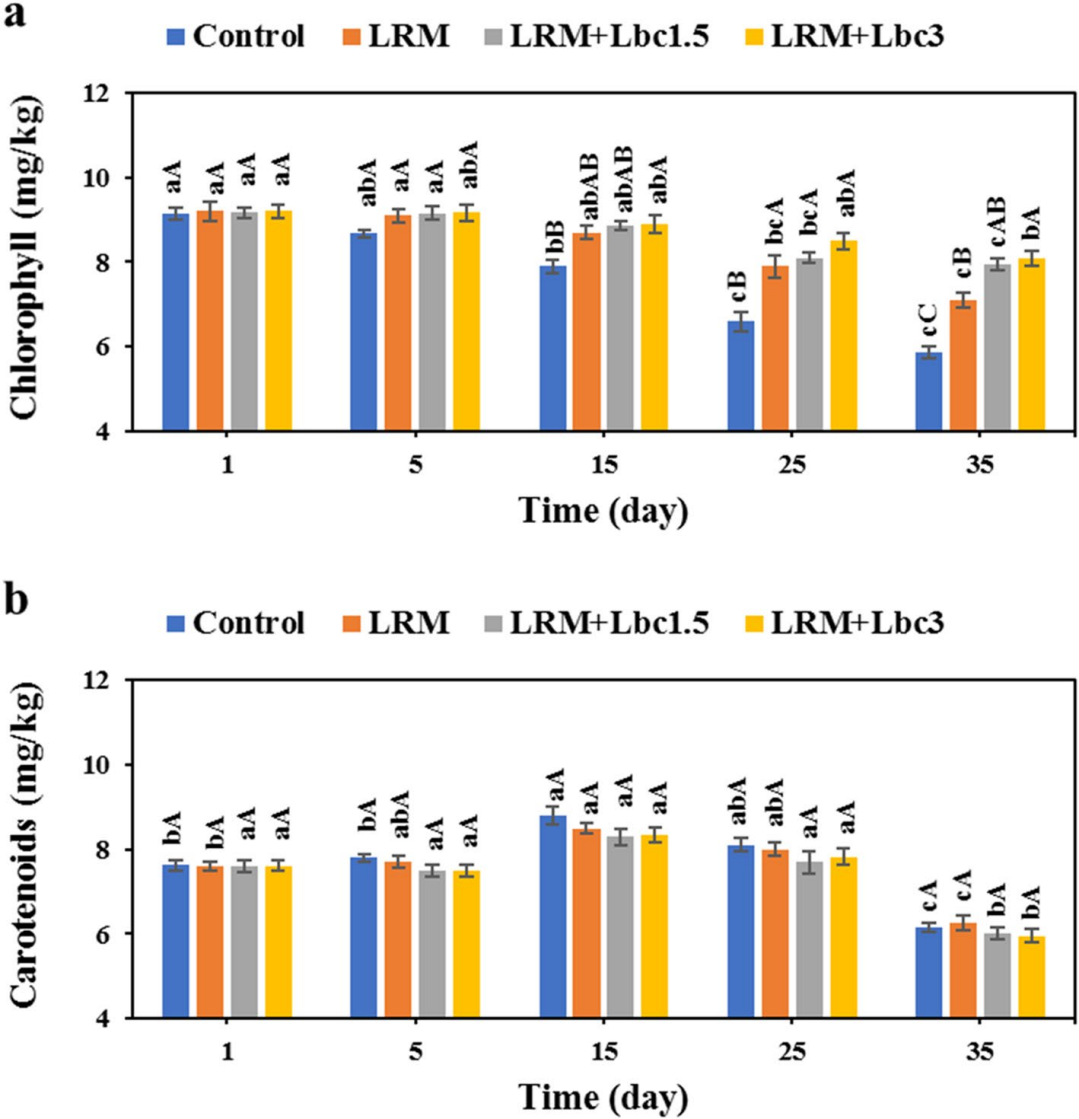
Changes in total chlorophyll content (**a**) and carotenoids content (**b**) of fresh in-hull pistachio samples coated with *Lallemantia royleana* mucilage (LRM) containing 1.5 × 10^8^ and 3 × 10^9^ CFU/ml *Lb. casei* XN18 (Lbc1.5 and Lbc3) during storage period. Significant small and capital letters indicate the effect of storage time and coating type on the response, respectively (p < 0.05).

The total content of chlorophylls of the pistachios decreased steadily over time (p < 0.05), and the highest and lowest changes in chlorophylls content were found in the control (9.15 to 5.85 mg/kg) and LRM + Lbc3 (9.20 to 8.10 mg/kg) during the storage period (Fig. 6a). The highest chlorophyll content is found in unripe green pistachios, and the content is decreased with ripening^37^ due to the activity of chlorophyll degrading enzymes including chlorophyllase, chlorophyll oxidase, and peroxidase^38^. The effect of storage time and edible coating on the total carotenoids content of the samples was also significant (Fig. 6b). The content of carotenoids increased manifestly as the storage time increased to 15 days; however, further increase in storage time decreased the carotenoids content. It could be generally stated that the content of total carotenoids in the samples during storage period followed the order: control (7.70 mg/kg) > LRM (7.61 mg/kg) > LRM + Lbc1.5 ~ LRM + Lbc3 (7.43 mg/kg). Lutein is known as the main carotenoid compound in the pistachio, and its level is dependent on the ripeness degree and origin of the nut^39^. Fruit ripening is accompanied by a decrease in chlorophyll level and an increase in carotenoid content, and these pigmentation changes of fruits facilitate the visual differentiation of ripe fruits at different stages^40^. In line with our study, Sheikhi et al.^41^ reported that the fresh pistachios under modified atmosphere packaging experienced a marked decrease in total chlorophylls and fluctuations in total carotenoids throughout the storage period. Moreover, the edible coatings have been found to delay the degradation of chlorophyll and carotenoid synthesis^42,43^. A high level of chlorophylls and lutein in this nut contribute to its antioxidant properties.

### TPC and antioxidant activity

**Figure 7:**
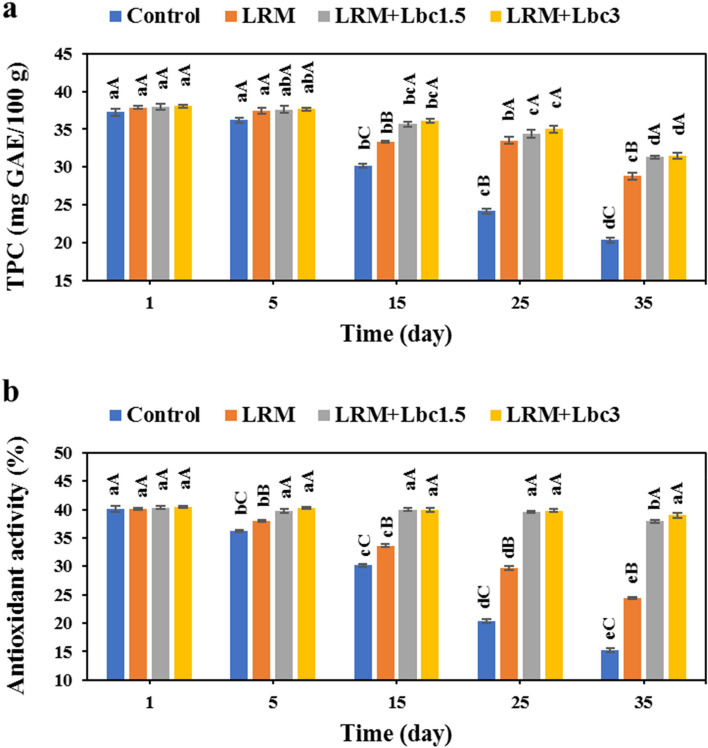
Changes in total phenol content-TPC (**a**) and antioxidant activity (**b**) of fresh in-hull pistachio samples coated with *Lallemantia royleana* mucilage (LRM) containing 1.5 × 10^8^ and 3 × 10^9^ CFU/ml *Lb. casei* XN18 (Lbc1.5 and Lbc3) during storage period. Significant small and capital letters indicate the effect of storage time and coating type on the response, respectively (p < 0.05).

The TPC of pistachio samples was significantly affected by the storage time and edible coating (Fig. 7a). All samples experienced a significant decrease in TPC as a function of storage time. In this context, the control, LRM, LRM + Lbc1.5, and LRM + Lbc3 pistachios showed a remarkable TPC decrease from 37.25 to 20.32, 37.85 to 28.80, 38.00 to 31.30, and 38.05 to 31.50 mg GAE/100 g over time, respectively. By the end of storage time, the samples coated with LRM, LRM + Lbc1.5, and LRM + Lbc3 had 1.42, 1.54, and 1.55 folds higher TPC compared to the control sample (p < 0.05). The decrease in TPC might be associated with the breakdown of phenolic compounds and the disintegration of cellular structures during the period of senescence^19^.

The capacity of fruits to act as antioxidants is linked to the presence of biomolecules such as phenols, flavonoids, carotenoids, chlorophylls, and vitamins. During postharvest storage, these biomolecules undergo oxidation, leading to an increase in free radicals and contributing to cellular aging^19^. A similar trend was observed for the antioxidant activity of the samples (Fig. 7b). Except for LRM + Lbc3, the antioxidant activity of pistachio samples decreased significantly as a function of storage time; the antioxidant activity of the control and LRM + Lbc3 on 35th storage day was 15.18 and 38.95%, respectively. The reduction of antioxidant activity during the storage period may be due to oxidation of phenolic compounds and the destruction of the cell structure during fruit senescence. These cause the release of oxidative and hydrolytic enzymes that may destroy the antioxidants^44^. However, the samples coated with LRM enriched with probiotic cells had markedly higher antioxidant capacity than the control and LRM pairs on 35 days of storage (p < 0.05). This could be due to the antioxidant activity of the strain^32^, lower oxygen permeability of the edible coating, lower fungal count (Fig. 2), and higher TPC (Fig. 6a) in such samples. It can be concluded that the presence of probiotic cells in the edible coating preserved antioxidant and phenolic compounds of pistachios during storage period. Similar findings were reported by Sheikhi et al.^41^ and Hashemi et al.^45^.

### Probiotic viability

**Figure 8:**
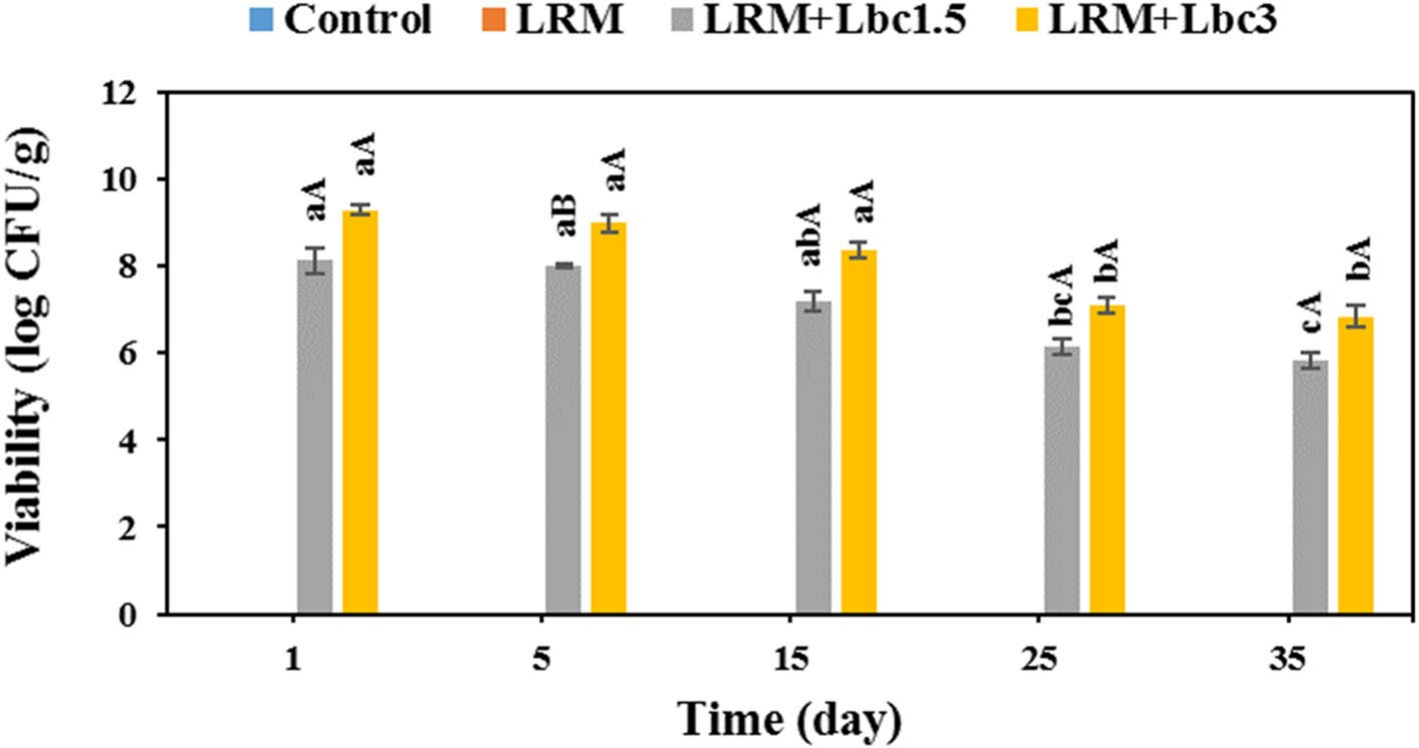
Changes in survivability of *Lb. casei* XN18 in fresh in-hull pistachio samples coated with *Lallemantia royleana* mucilage (LRM) containing 1.5 × 10^8^ and 3 × 10^9^ CFU/ml *Lb. casei* XN18 (Lbc1.5 and Lbc3) during storage period. Significant small and capital letters indicate the effect of storage time and coating type on the response, respectively (p < 0.05).

The number of viable probiotics on the LRM + Lbc coated samples during storage period is provided in Fig. 8. The viable cell counts of *Lb. case* XN18 in the samples decreased markedly as the storage time prolonged to 35 days (p < 0.05). There are several factors that influence the efficiency of probiotic addition into food, including the inoculum cell concentration and viability during storage^46^. In this context, higher probiotic addition into edible coating led to greater viable cells on the coated pistachios. There is no universal recommendation for the minimum number of viable probiotics in food for health benefits, but most food manufacturers follow the recommended level of 6 log CFU/g^47^. The sample coated with LRM + Lbc3 had a probiotic number above the recommended level (6.85–9.29 log CFU/g) throughout the storage time, and it can therefore be considered a probiotic food product. However, the LRM + Lbc1.5 coated pistachio sample was acceptable in the term of probiotic level until 25 days of storage. Similarly, edible coatings were found to efficiently protect probiotic cells on fruits/vegetables during storage period^48–50^.

### Sensory characterization

**Figure 9:**
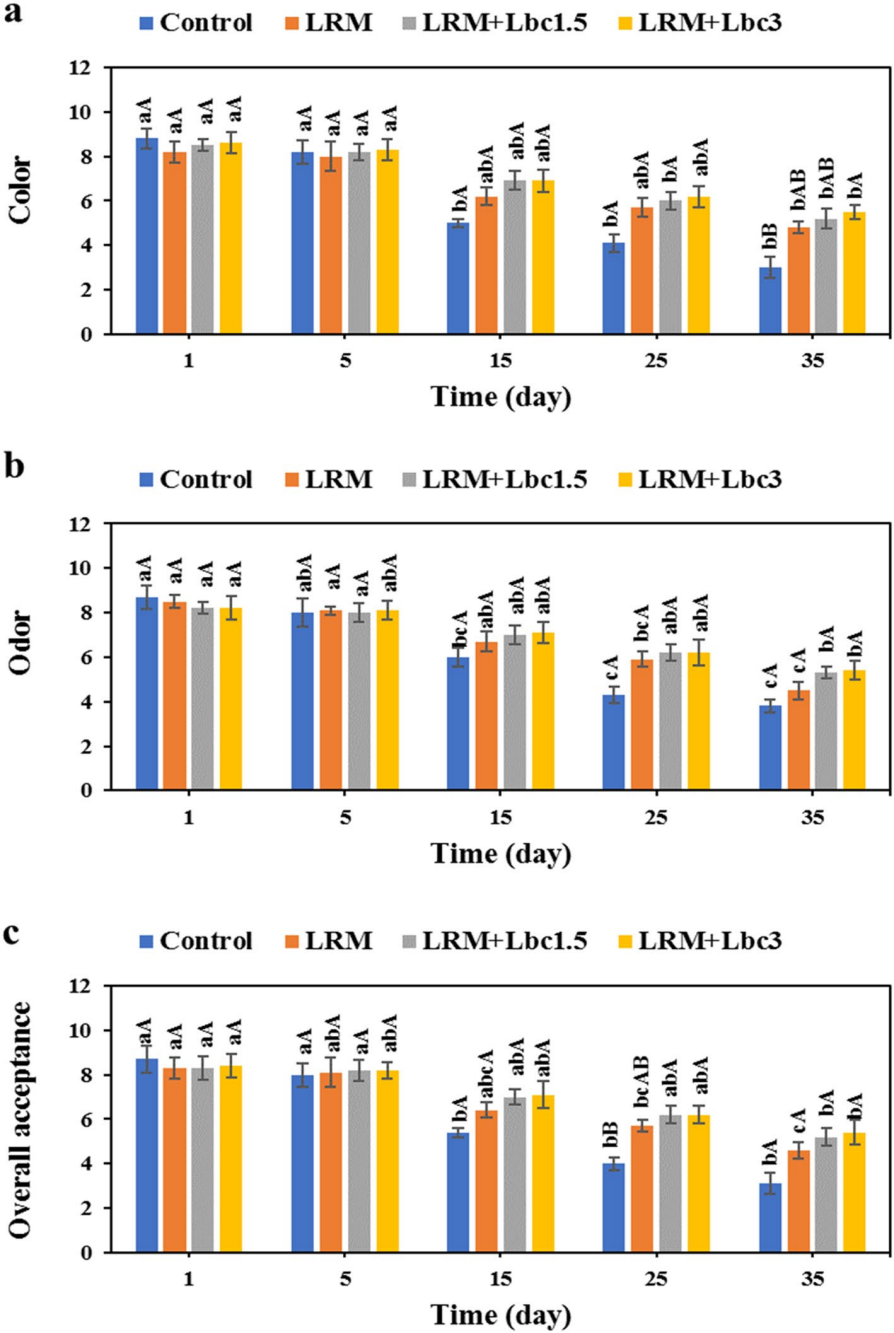
Changes in color (**a**), odor (**b**), and overall acceptance (**b**) of fresh in-hull pistachio samples coated with *Lallemantia royleana* mucilage (LRM) containing 1.5 × 10^8^ and 3 × 10^9^ CFU/ml *Lb. casei* XN18 (Lbc1.5 and Lbc3) during storage period. Significant small and capital letters indicate the effect of storage time and coating type on the response, respectively (p < 0.05).

All samples experienced a significant decrease in color and odor during storage time (Fig. 9a,b); however, the changes in sensory properties of pistachios coated with LRM + Lbc were remarkably lower compared to the control sample. This result is supported by the total chlorophylls and carotenoids content of the samples (Fig. 6). The overall acceptance of samples showed a similar trend (Fig. 9c). The control, LRM, LRM + Lbc1.5, and LRM + Lbc3 pistachios showed an overall acceptance score reduction of 5.6, 3.7, 3.1, and 3 score over time, respectively. By the end of storage time, LRM, LRM + Lbc1.5, and LRM + Lbc3 coated samples had 32.61, 40.38, and 42.59% higher overall acceptance scores compared to the control sample. This means that pistachios coated with probiotic-enriched edible coating were greatly accepted by panelists. Khodaei and Hamidi-Esfahani^18^ reported that, during a storage period of 10 days, strawberries coated with carboxymethyl cellulose edible coating enriched with *L. plantarum* showed acceptable color, flavor, taste, texture, and overall acceptance. It is important to maintain consumer acceptance of probiotic supplemented foods, because consumers tend not to consume functional foods if the added ingredients cause off-flavors, regardless of the health benefits^16^.

### Gaussian process regression (GPR) model

**Figure 10:**
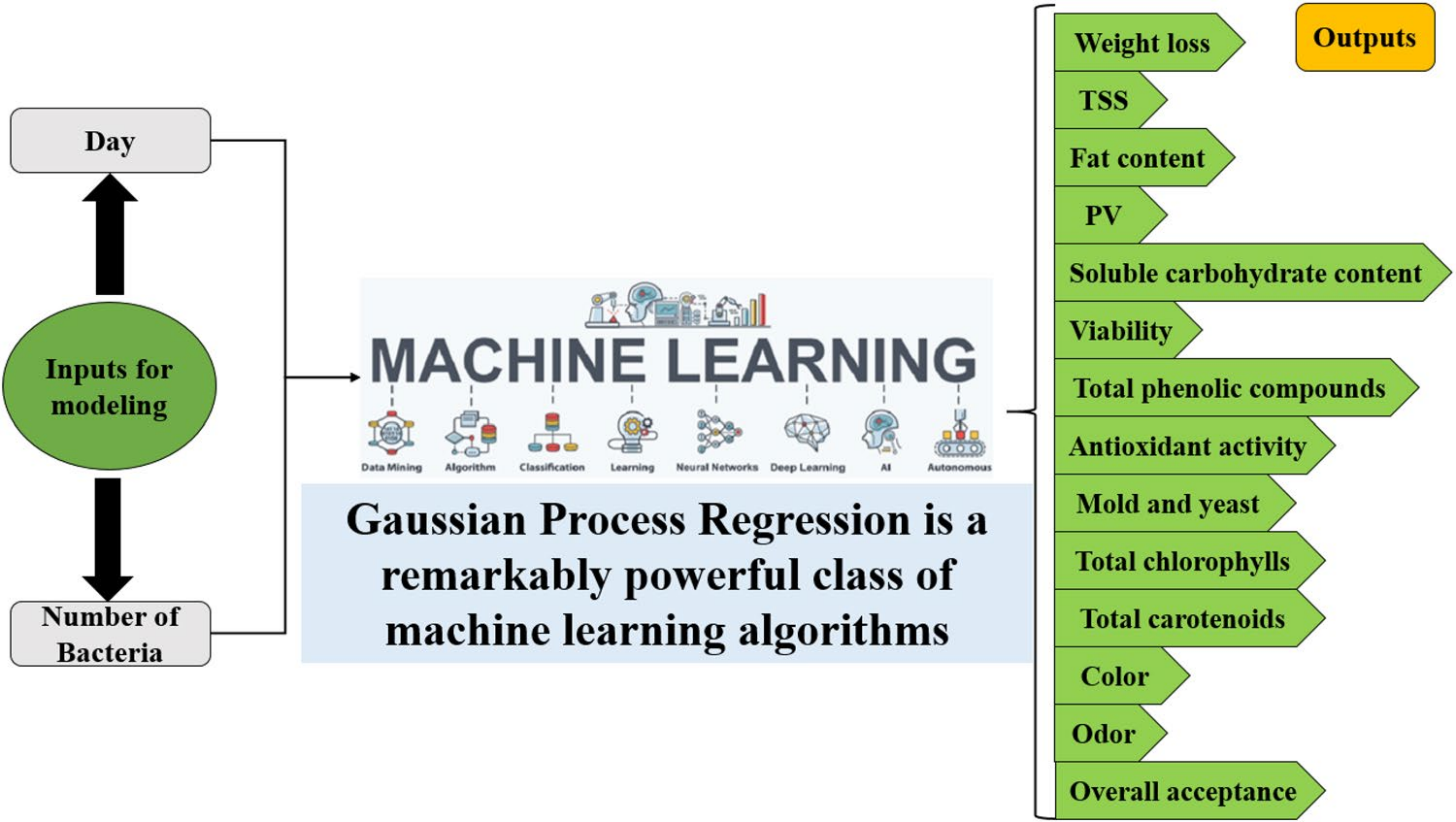
All inputs and outputs used in GPR modeling.

In this step, GPR model was applied for predicting the all 14 parameters including: weight loss, TSS, Fat content, PV, Soluble carbohydrate content, Viability, Total phenolic compounds, Antioxidant activity, Mold and yeast, Total chlorophylls, Total carotenoids, Color, Odor and Overall acceptance (Fig. 10).

Table 1 shows the results of GPR model based on the three general statistical indexes in training, test and total phases. The results indicated that GPR can predict the output data with good accurate (MAPE was between 0.1 to 8.4%). The study findings reveal that the fat content exhibits the lowest MAPE of 0.1%, while mold and yeast have the highest MAPE of 8.4%. Optimal results are achieved when the linear equation between the actual and predicted values by the model demonstrates the highest coefficient of determination, the narrowest width from the origin, and a slope close to one. Importantly, the GPR model exhibits a high correlation coefficient in both the training and test stages. Additionally, the regression relationships observed in different stages show the narrowest width from the origin and a slope close to one. This indicates that the GPR model can be reliably utilized as a useful method to predict all 14 studied parameters in similar cases, effectively reducing the need for costly laboratory tests. The outcomes of the GPR model, utilizing three general statistical indexes in the training, test, and total phases, are presented in Table 1. The results clearly indicate that the GPR model achieves accurate predictions of the output data, as evidenced by the MAPE values ranging from 0.1 to 8.4%. Notably, the fat content exhibits the lowest MAPE of 0.1%, while mold and yeast have the highest MAPE of 8.4%. The optimal performance of the GPR model is observed when the linear equation between the actual and predicted values by the model demonstrates the highest coefficient of determination, the narrowest width from the origin, and a slope close to one ($pv = 1.00dv + 0$). The study further reveals that the GPR model exhibits a strong correlation coefficient in both the training and test stages. Moreover, the regression relationships observed in various stages show the narrowest width from the origin and a slope close to one. These findings establish the GPR model as a reliable and valuable approach for predicting all 14 studied parameters in similar cases, thereby reducing the reliance on expensive laboratory tests.

**Table 1:** Statistical analysis of GPR models for predicting all the 12 parameters at training, test and total phases.

Output parameter	Train			Test			Total		
	RMSE	MAPE	R^2^	RMSE	MAPE	R^2^	RMSE	MAPE	R^2^
Weight loss	0.08	4.46	0.99	0.15	2.39	0.98	0.10	4.04	0.99
	Pv = 0.99 × Av + 0.01			Pv = 1.13 × dv-0.69			Pv = 1.01 × dv-0.01		
TSS	0.22	1.37	0.99	0.33	2.08	0.96	0.24	1.51	0.99
	Pv = 0.99 × Av + 0.13			Pv = 0.97 × Av + 0.31			Pv = 0.99 × Av + 0.10		
Fat content	0.09	0.11	0.97	0.07	0.09	0.98	0.08	0.10	0.98
	Pv = 0.95 × Av + 2.79			Pv = 2.12 × Av-2.65			Pv = 0.96 × Av + 2.50		
PV	0.01	0.11	0.99	0.01	9.34	0.99	0.01	1.95	0.99
	Pv = 0.99 × Av + 0.01			Pv = 0.73AV + 0.04			Pv = 0.99 × Av + 0.01		
Carbohydrate content	0.01	0.07	0.99	0.05	0.59	0.99	0.02	0.18	0.99
	Pv = 0.99 × Av + 0.02			Pv = 0.90 × Av + 0.86			Pv = 0.97 × Av + 0.27		
Viability	0.15	0.28	0.99	0.12	0.04	0.99	0.15	0.33	0.99
	Pv = 0.99 × Av + 0.04			Pv = 0.99 × Av + 0.01			Pv = 0.99 × Av + 0.03		
Total phenolic compounds	0.44	1.04	0.97	0.21	0.50	0.88	0.41	0.94	0.98
	Pv = 0.95 × Av + 1.88			Pv = 1.50 × Av-18.67			Pv = 0.97 × Av + 1.11		
Antioxidant content	0.26	0.62	0.99	0.17	0.43	0.96	0.25	0.58	0.99
	Pv = 0.98 × Av + 0.63			Pv = 1.02 × Av-0.72			Pv = 0.98 × Av + 0.62		
Mold and yeast	0.17	7.52	0.96	0.29	11.92	0.81	0.20	8.40	0.94
	Pv = 0.91 × Av + 0.16			Pv = 0.90 × Av + 0.21			Pv = 0.91 × Av + 0.16		
Total chlorophylls	0.07	0.58	0.99	0.04	0.36	0.97	0.06	0.54	0.98
	Pv = 0.97 × Av + 0.27			Pv = 0.48 × Av + 4.78			Pv = 0.98 × Av + 0.14		
Total carotenoids	0.09	0.88	0.98	0.22	2.54	0.98	0.12	1.22	0.98
	Pv = 0.98 × Av + 0.15			Pv = 1.06 × Av-0.63			Pv = 1.00 × Av-0.07		
Color	0.02	0.23	0.99	0.21	2.26	0.98	0.09	0.63	0.99
	Pv = 0.99 × Av + 0.01			Pv = 1.00 × Av-0.21			Pv = 0.99 × Av + 0.01		
Odor	0.04	0.39	0.99	0.45	7.21	0.97	0.20	1.75	0.98
	Pv = 0.99 × Av + 0.03			Pv = 0.76 × Av + 1.62			Pv = 0.90 × Av + 0.66		
Overall acceptances	0.04	0.54	0.99	0.32	3.97	0.90	0.15	1.23	0.98
	Pv = 0.99 × Av + 0.02			Pv = 0.95 × Av + 0.38			Pv = 0.99 × Av + 0.06		

*Pv* predicted values, *Av* actual values.

## Conclusions

This study is the first to examine the effects of active LRM coatings containing *Lb. casei* XN18 on fresh in-hull pistachio’s physicochemical, microbial, and sensory properties during 35 days of storage at 4 °C. The probiotic-loaded LRM coatings, particularly those loaded with 3 × 10^9^ CFU/mL probiotic cells, were more effective in maintaining physicochemical, microbial, and sensory properties of pistachio samples compared to the control counterpart. However, there is a need for further investigation into the long-term efficacy of active coatings containing probiotic isolates on the shelf-life of pistachios. Additionally, comparing various coatings for pistachios and other nuts based on biopolymers and other probiotic strains can be recommended to discover the most effective and economical active coating. GPR model was used for predicting some of output parameters. The results indicated that GPR can be used with high level of accuracy for predicting the output even when the number of available data is small.

## Data Availability

All data relevant to the study are included in the article.
